# Millets for Next Generation Climate-Smart Agriculture

**DOI:** 10.3389/fpls.2017.01266

**Published:** 2017-07-18

**Authors:** Tirthankar Bandyopadhyay, Mehanathan Muthamilarasan, Manoj Prasad

**Affiliations:** National Institute of Plant Genome Research New Delhi, India

**Keywords:** millets, climate-smart agriculture, foxtail millet, crop improvement, food security, nutrition security

## Millets: promising crops for the TWENTY-first century

Panicoids (subfamily: panicoideae) are a group of C_4_ grasses, which include agronomically important crops such as sorghum and maize, bioenergy feedstocks including sugarcane and miscanthus, nutri-cereals such as millets, and biofuel crops including switchgrass, napier grass and guinea grass. Among these, millets are known for their climate-resilient features including adaptation to a wide range of ecological conditions, less irrigational requirements, better growth and productivity in low nutrient input conditions, less reliance on synthetic fertilizers, and minimum vulnerability to environmental stresses (Kole et al., 2015). Also, millets are nutritionally superior to other major cereals as they are rich in dietary fibers, resistant starches, vitamins, essential amino acids, storage proteins and other bioactive compounds (Amadou et al., 2013). These attributes have made millets a crop of choice for cultivation in arid and semi-arid regions of the world; however, the less attempt has been made to study the climate-resilient features of millets compared to other major cereals. Among millets, foxtail millet (*Setaria italica*) and its wild progenitor, green foxtail (*S. viridis*) are extensively studied since they are considered as models for studying the traits related to C_4_ photosynthesis, stress biology, and bioenergy characteristics (Muthamilarasan and Prasad, 2015). The availability of genome sequence information of these two species (Bennetzen et al., 2012; Zhang et al., 2012) has unlocked the wealth of information pertaining to stress tolerance and biofuel characteristics. It has also expedited the development of large-scale genomic resources for crop improvement. On the other hand, studies on other millets are still in their infancy. The challenge to feed the ever-growing population with a healthy balanced diet and the threats faced by agricultural crops due to changing climate highlight the immediate requirement to exploit the beneficial attributes of millets. This could be utilized for the improvement of millets *per se* as well as other related grass species. The extensive gene-level synteny shared between the grass genomes would facilitate the transfer and introgression of useful genes, alleles and quantitative trait loci (QTL) of agronomic importance identified in millets to other major cereals. In the above context, this article advocates for initiating extensive research on millets to dissect their agronomic, nutritional as well as stress tolerance traits and develop strategies to transfer the useful traits to cultivated major cereals such as rice, wheat, maize, and sorghum.

## Traits contributing climate-resilience to millets

Millets possess several morpho-physiological, molecular and biochemical characteristics which confer better tolerance to environmental stresses than major cereals. Primarily, the short life-cycle of millets assists in escaping from stress as they require 12–14 weeks to complete their life-cycle (seed to seed) whereas rice and wheat requires a maximum of 20–24 weeks. However, the prevalence of stress conditions and their consequences are circumvented by several traits such as short stature, small leaf area, thickened cell walls, and the capability to form dense root system (Li and Brutnell, 2011). Also, the C_4_ photosynthetic trait is highly advantageous to millets. In the C_4_ system, carbon dioxide (CO_2_) is concentrated around ribulose-1,5-bisphosphate carboxylase/oxygenase (RuBisCO), which in turn suppresses ribulose 1,5-bisphosphate (RuBP) oxygenation and photorespiration (Aubry et al., 2011). Thus, C_4_ mechanism enhances the concentration of CO_2_ in bundle sheath, which suppresses photorespiration (around 80%) depending on the temperature and increases the *in planta* catalytic activity of RuBisCO (Sage et al., 2011). Since RuBisCO of C_4_ plants works at elevated CO_2_ levels, millets have enhanced photosynthetic rates at warm conditions and confers immediate water use efficiency (WUE) and nitrogen use efficiency (NUE) which are ~1.5 to 4-fold higher than C_3_ photosynthesis (Sage and Zhu, 2011). For instance, foxtail millet requires just 257 g of water to produce a dry biomass of 1 g, whereas maize and wheat require 470 and 510 g, respectively (Li and Brutnell, 2011). In addition to conferring WUE and NUE, C_4_ photosynthesis provides secondary benefit to millets including improved growth and ecological enactment in warm temperatures, enhanced flexible allocation patterns of biomass and reduced hydraulic conductivity per unit leaf area (Sage and Zhu, 2011). These attributes of millets make them next-generation crops holding the potential for research to explore the climate-resilient traits and exploit the information for the improvement of major cereals. One such effort undertaken so far is the engineering of C_4_ traits in rice using millet as models; however, through the realization of stress tolerance potential of millets is imperative for expedited progress in developing climate-resilient crop species.

## Millets as the model for stress biology

The exceptional tolerance of millets toward diverse abiotic stresses including drought, salinity, light and heat makes them a tractable system to study their stress-responsive traits at the cellular, molecular and physiological levels. Several morpho-physiological and biochemical studies in millets have shown their stress adaptation strategies. For example, Bidinger et al. (2007) have shown that pearl millet adjusts flowering phenology according to the pattern of rainfall. Balsamo et al. (2006) observed an increase in leaf tensile strength in teff during drought, and in little millet, an increase in root length was reported by Ajithkumar and Panneerselvam (2014). Similarly, increase in biochemical activities such as enhanced levels of antioxidants, reactive oxygen species and their scavenging enzymes, enzyme activity of catalase and superoxide, and synthesis of osmolytes and other stress-related proteins has been reported in response to abiotic stresses in foxtail millet (Lata et al., 2011), little millet (Ajithkumar and Panneerselvam, 2014) and teff (Smirnoff and Colombe, 1988). van der Weerd et al. (2001) showed the dynamics of membrane permeability for water in pearl millet in comparison to maize for achieving better water status during osmotic stress. In addition, several novel genes, alleles and QTLs have been identified in millets whose functional characterization has revealed their roles in conferring stress tolerance (Table 1). Compared to other millets, foxtail millet has been studied extensively, and several genetic and genomic resources have been developed (Muthamilarasan and Prasad, 2015). Whole genome sequencing of foxtail millet and comparison of gene families among 15 sequenced plant genomes showed that 1517 genes were specific only to foxtail millet (Zhang et al., 2012). Among this, 586 genes were annotated as “response to water,” which could be playing significant roles in conferring drought and dehydration stresses, thus facilitating the adaptation of this crop to arid and semi-arid zones. The genes involved in C_4_ pathway namely, carbonic anhydrase (CAH), malate dehydrogenase (MDH), malic enzyme (ME), phosphoenolpyruvate carboxylase (PEPC), phosphoenolpyruvate carboxylase kinase (PPCK) and pyruvate orthophosphate dikinase (PPDK) were also identified and compared to that of sorghum, maize, rice and *Brachypodium*. The study showed that foxtail millet has a higher number of MDH (7 genes) and PPDK (3 genes) than other crops. Zhang et al. (2012) have also performed phylogenetic and evolutionary analysis of CAH homologs among all the five grass genomes, which showed that *Ft_CA1* was highly expressed in the mesophyll, and this could be a potential candidate for studying C_4_ pathway in foxtail millet. Despite this progress, studies providing insights into the molecular machinery underlying stress tolerance is largely lacking in millets. In addition, knowledge on the genetic determinants of stress tolerance identified through association mapping and biparental mapping is limited. In this context, extensive phenotypic screening to observe the natural genetic variations in stress tolerance across diverse millet germplasms is greatly needed to fully harness the underlying genetic potential through conventional/molecular breeding approaches and transgenic technologies. This is required to facilitate crop improvements in millets and non-millet crops in the wake of increased desertification and salinity of the farmlands due to climate change.

**Table 1:** Summary of genes identified and characterized in different millets for their roles in conferring tolerance to abiotic stresses.

**Millet**	**Gene**	**Nature of study**	**References**
Foxtail millet	Argonaute protein 1 encoding gene	AGO1b has been shown to regulate stress response in foxtail millet	Liu et al., 2016
	Abscisic acid stress ripening gene (ASR)	Overexpression of ASR1 in tobacco confers tolerance to drought and oxidative stress	Feng et al., 2016
	Autophagy-related gene (ATG)	Overexpression of ATG8a in *Arabidopsis* confers tolerance to nitrogen starvation and drought stress	Li et al., 2015
	Late embryogenesis abundant protein (LEA)	Overexpression of LEA14 in *Arabidopsis* and foxtail millet confers tolerance to salt, osmotic and drought stress	Wang et al., 2014
	ABA-responsive DRE-binding protein (ARDP)	Overexpression of ARDP in *Arabidopsis* and foxtail millet confers tolerance to salt and drought stress	Li et al., 2014
	WD-40	Identification of the association of WD40 in dehydration stress-responsive pathway	Mishra et al., 2012
	Acetyl-CoA carboxylase	Overexpression of Acetyl-CoA carboxylase in maize confers resistance to sethoxydim herbicide	Dong et al., 2011
	Dehydration-responsive element-binding protein 2 (DREB2)	Cloning and characterization of DREB2 showed its role in conferring dehydration tolerance	Lata et al., 2011
	NAC transcription factor	Cloning and characterization of NAC078 showed its role in conferring salinity tolerance	Puranik et al., 2011
	Si69	Overexpression of wheat aluminum induced protein (Wali) domain containing protein in *Arabidopsis* confers aluminum tolerance	Zhao et al., 2009
	Aldose reductase	Identification of the association of respective genes in salinity stress-responsive pathway	Veeranagamallaiah et al., 2009
	Glutamine synthetase		Veeranagamallaiah et al., 2007
	Pyrroline-5-carboxylate reductase		
	12-oxophytodienoic acid reductase (OPR1)	Cloning and characterization of OPR1 showed hormone-independent role of OPR1 in conferring drought tolerance	Zhang et al., 2007
	Photosystem II D_1_protein	Identification of the association of Photosystem II D_1_protein-encoding gene in conferring atrazine resistance	Jia et al., 2007
	Phospholipid hydroperoxide glutathione peroxidase (PHGPX)	Cloning and characterization of PHGPX showed its association in conferring salinity tolerance	Sreenivasulu et al., 2004
Finger millet	Phosphate transporters (Pt)	Cloning and characterization of four Pt genes which showed their involvement in Pi stress	Pudake et al., 2017
	NAC transcription factor	Overexpression of NAC67 gene in rice confers tolerance to salinity and drought stress	Rahman et al., 2016
		Overexpression of NAC1 gene in tobacco confers tolerance to different abiotic stresses	Ramegowda et al., 2012
	bHLH transcription factor	Overexpression of bHLH57 gene in tobacco confers tolerance to salinity, oxidative and drought stress	Babitha et al., 2015
	Dehydrin7	Overexpression of Dehydrin7 gene in tobacco confers tolerance to drought stress	Singh et al., 2015
Pearl millet	Glutathione reductase	Identification and characterization of genes and their families highlighted their putative involvement in stress-responsive pathways	Achary et al., 2015
	Dehydroascorbate reductase		Pandey et al., 2014
	Late embryogenesis abundant protein (LEA)		Reddy et al., 2012
	β-carbonic anhydrase		Kaul et al., 2011
	Ascorbate peroxidase		Reddy et al., 2009
	Heat shock factor		
	Voltage-dependent anion channel (VDAC)	Structural and functional characterization of VDAC along with heterologous over-expression in yeast which showed tolerance to several abiotic stresses	Desai et al., 2006

## Genetic and genomic resources available in millets

Genetic and genomic resources are imperative for the improvement of any crop species, where genetic resources serve as primary input for breeding while genomic resources facilitate efficient characterization of genetic resources and their subsequent utilization in the identification of useful genes, alleles, and QTLs for crop improvement. In the case of millets, ample genetic resources are available (Goron and Raizada, 2015; Saha et al., 2016); however, the information available on genomic resources including molecular markers and physical/genetic maps are scarce as compared to major cereals. Availability of genome sequence information of foxtail millet has facilitated the development of several high-throughput genome-wide molecular markers (Jia et al., 2013; Kumari et al., 2013; Pandey et al., 2013; Muthamilarasan et al., 2014; Yadav et al., 2014, 2015; Zhang et al., 2014) and integrated marker databases (Muthamilarasan and Prasad, 2015). These resources would be inevitable for several large-scale genotyping studies including genetic diversity analysis, construction of high-density physical as well as genetic-linkage maps, and mapping of QTLs related to nutritional traits. One such example for utilization of these resources in genomics-assisted breeding (GAB) was reported by Gupta et al. (2014). In this study, association mapping of 20 yield-contributing agronomic traits among 200 foxtail millet accessions was performed, and eight microsatellite markers associated with nine different agronomic traits were identified, which contribute up to 25% of the phenotypic variation (Gupta et al., 2014). These trait-associated SSR markers will be useful for identification of genes/QTLs regulating the agronomic as well as nutritional traits, and eventually for marker-assisted genetic enhancement of foxtail millet *per se* and millets and cereals.

Recently, GAB has gained momentum in the arena of crop improvement as it implicates the next-generation genome analysis platforms and conventional as well as molecular breeding strategies for crop improvement. The scope for development of genomic resources for GAB in foxtail millet is relatively higher than other millets owing to the availability of genome sequence information. Moreover, the advent of next-generation genome sequencing (NGS) has enabled the development of high-throughput molecular markers in other millet crops. In finger millet, the transcriptome of high and low seed calcium genotypes was sequenced, and thousands of simple sequence repeat (SSR) containing microsatellite markers were identified (Kumar et al., 2015). Similarly, novel SSR markers were developed through sequencing the genome of allotetraploid tef (*Eragrostis tef*) (Cannarozzi et al., 2014). This information on the genome as well as genome-wide markers coupled high-throughput approaches including genotyping-by-sequencing (GBS) and genome-wide association mapping studies (GWAS) potentiate the discovery of novel genes/alleles or QTLs responsible for nutritional traits (Varshney et al., 2014; Muthamilarasan et al., 2016). Considering the importance of whole-genome sequence information, genome sequencing of few other millets including pearl millet and finger millet is underway.

## Conclusion

It is realized that millets hold great promise for food security and nutrition amid ever-increasing agricultural costs, climate change and burgeoning mouths to feed worldwide. They are nutritious, possess additional health benefits, requires significantly fewer input costs for cultivation and are naturally tolerant to most biotic and abiotic stresses. These features accentuate millets as crops of choice for the world population amid growing concerns about climate change. Given the same, there is a growing need to investigate the natural genetic variation across their diverse germplasms to exploit them for crop improvement with regard to many agronomically and nutritionally important traits. With the advent of NGS technologies and high-throughput GWAS platforms, identification of candidate genes/alleles/QTLs regulating such traits is possible at a pace and precision not contemplated before, which in turn would facilitate the development of breeding lines for crop improvement. Moreover, a renewed focus on millets has important implications for the improvement of cereals and bioenergy grasses given their common ancestry from poaceae family and the presence of significant synteny between the genomes.

## Author contributions

MP and TB conceived the idea and drafted the opinion article. MM improved the manuscript and provided revisions to the manuscript. All the authors have read and approved the final version of the manuscript.

### Conflict of interest statement

The authors declare that the research was conducted in the absence of any commercial or financial relationships that could be construed as a potential conflict of interest.
